# Supplementary material for: Analysis of genes and underlying mechanisms involved in foam cells formation and atherosclerosis development
Source: PeerJ. 2020 Nov 17;8:e10336. doi: 10.7717/peerj.10336 (PMC7678445; doi:10.7717/peerj.10336)
Supplement: Table S1 [file peerj-08-10336-s001.doc]

Table S1 GO-BP and KEGG pathway analysis of SMCs-FC-related genes

| Category | Term | Count | GeneRatio | PValue | Genes |
| --- | --- | --- | --- | --- | --- |
| GO-BP | GO:0007015~actin filament organization | 10 | 0.023148 | 5.4E-05 | MYO1B, TPM2, TPM1, FAT1, RAC2, LMOD1, WAS, EMP2, TMSB10, DBN1 |
| GO-BP | GO:0019886~antigen processing and presentation of exogenous peptide antigen via MHC class II | 10 | 0.023148 | 0.000362 | DYNC1I2, CD74, DYNC1LI2, KLC2, FCER1G, AP1B1, AP2S1, CTSD, RILP, DYNLL2 |
| GO-BP | GO:0050852~T cell receptor signaling pathway | 12 | 0.027778 | 0.000898 | NFKBIA, PIK3CA, PSME1, WAS, PTPRJ, THY1, PAG1, PSMB8, PSMB10, TNFRSF21, PSMB9, SKP1 |
| GO-BP | GO:0006915~apoptotic process | 26 | 0.060185 | 0.00265 | CLIC4, SH3KBP1, EI24, SEMA3A, PRUNE2, CDCA7, FXR1, PYCARD, PIM3, MAP3K8, JAK2, ZNF385A, CTSC, MAP3K1, DAPK1, AKTIP, CKAP2, SERPINB9, RHOB, NFKBIA, RNF144B, PLSCR1, CDK1, TAX1BP1, CHI3L1, TNFRSF21 |
| GO-BP | GO:0031214~biomineral tissue development | 5 | 0.011574 | 0.002773 | ECM1, TMEM119, SPP1, ENPP1, FAM20A |
| GO-BP | GO:0002479~antigen processing and presentation of exogenous peptide antigen via MHC class I, TAP-dependent | 7 | 0.016204 | 0.003949 | PSME1, CYBA, HLA-F, B2M, PSMB8, PSMB10, PSMB9 |
| GO-BP | GO:0007035~vacuolar acidification | 4 | 0.009259 | 0.004101 | ATP6V0B, CLIC4, DMXL2, TCIRG1 |
| GO-BP | GO:0051603~proteolysis involved in cellular protein catabolic process | 6 | 0.013889 | 0.005699 | CTSZ, PSMB8, CTSC, PSMB10, PSMB9, CTSB |
| GO-BP | GO:0007266~Rho protein signal transduction | 6 | 0.013889 | 0.006783 | ROCK1, ROCK2, PECAM1, RHOG, AGTR1, RHOB |
| GO-BP | GO:0007165~signal transduction | 42 | 0.097222 | 0.008535 | GRIA2, ECM1, ROCK1, PDE1A, ADCY3, SECTM1, LSP1, RASAL3, C3, PYCARD, IRAK1, GNG5, SH3BP2, TP53BP2, RAC2, LPXN, JAK2, SH2B3, CLIC1, PAG1, PRKG1, MPP1, CD74, PRKAB2, PRKCH, PPP1R12A, CHKA, IFNGR1, DAPK1, IGFBP4, NFAM1, PLAUR, RASSF8, LNPEP, GNG12, IGF2R, TLR1, TYROBP, PECAM1, PKIG, IL7R, TNFRSF21 |
| GO-BP | GO:0045022~early endosome to late endosome transport | 4 | 0.009259 | 0.008564 | AKTIP, EMP2, RILP, RAB5A |
| GO-BP | GO:0006939~smooth muscle contraction | 4 | 0.009259 | 0.008564 | SMTN, ROCK1, ROCK2, MYLK |
| GO-BP | GO:0007040~lysosome organization | 5 | 0.011574 | 0.010499 | ABCA1, CLN3, AKTIP, TPP1, LYST |
| GO-BP | GO:0042059~negative regulation of epidermal growth factor receptor signaling pathway | 5 | 0.011574 | 0.010499 | SH3KBP1, AP2S1, PTPRJ, STAM2, PTPN2 |
| GO-BP | GO:0048841~regulation of axon extension involved in axon guidance | 3 | 0.006944 | 0.011139 | PLXND1, SEMA3A, PLXNC1 |
| GO-BP | GO:1903140~regulation of establishment of endothelial barrier | 3 | 0.006944 | 0.011139 | PPP1R12A, ROCK1, ROCK2 |
| GO-BP | GO:0006491~N-glycan processing | 4 | 0.009259 | 0.011548 | MAN1A2, MAN2A1, EDEM2, MAN1C1 |
| GO-BP | GO:0045861~negative regulation of proteolysis | 4 | 0.009259 | 0.013237 | CLN3, CSTB, CSTA, PLAT |
| GO-BP | GO:0006468~protein phosphorylation | 20 | 0.046296 | 0.014216 | PRKAB2, PRKCH, MAP3K1, DMPK, ROCK1, ROCK2, DAPK1, ILK, TYK2, MYLK, CDK8, IRAK1, PIK3CA, STK38L, PIM3, MAP3K8, JAK2, JAK3, MAP3K11, PRKG1 |
| GO-BP | GO:0034113~heterotypic cell-cell adhesion | 4 | 0.009259 | 0.015059 | ITGA4, PERP, ITGAX, ITGA7 |
| GO-BP | GO:0007049~cell cycle | 12 | 0.027778 | 0.016021 | PCNP, EID1, USP16, TP53BP2, PIM3, MAP3K8, ZMYND11, GNAI1, CDC73, MAPK13, CDKN3, PPP1CA |
| GO-BP | GO:0000902~cell morphogenesis | 6 | 0.013889 | 0.016422 | SS18, LST1, FRY, IL7R, PSMB10, SIDT2 |
| GO-BP | GO:0030163~protein catabolic process | 5 | 0.011574 | 0.016443 | CLN3, PPT1, TPP1, LNPEP, CTSD |
| GO-BP | GO:0035556~intracellular signal transduction | 18 | 0.041667 | 0.017971 | SS18, PRKCH, DAPK1, NFAM1, PLCL1, ADCY3, TYK2, MAPK13, LAT2, SDCBP, EFS, TYROBP, PLCB4, STK38L, JAK2, SH2B3, JAK3, PAG1 |
| GO-BP | GO:0035456~response to interferon-beta | 3 | 0.006944 | 0.018499 | IFNAR2, IFITM1, PLSCR1 |
| GO-BP | GO:0002480~antigen processing and presentation of exogenous peptide antigen via MHC class I, TAP-independent | 3 | 0.006944 | 0.018499 | LNPEP, HLA-F, B2M |
| GO-BP | GO:0021785~branchiomotor neuron axon guidance | 3 | 0.006944 | 0.018499 | PLXND1, SEMA3A, PLXNC1 |
| GO-BP | GO:0060337~type I interferon signaling pathway | 6 | 0.013889 | 0.018618 | IFNAR2, IFITM1, TYK2, HLA-F, PSMB8, IFIT3 |
| GO-BP | GO:0030155~regulation of cell adhesion | 5 | 0.011574 | 0.019306 | PPP1CB, PPP1R12A, ROCK1, ROCK2, PTPRJ |
| GO-BP | GO:0015949~nucleobase-containing small molecule interconversion | 4 | 0.009259 | 0.021335 | RRM2, CMPK1, CTPS2, GLRX |
| GO-BP | GO:0006461~protein complex assembly | 8 | 0.018519 | 0.021795 | CD74, TSPAN4, CDK1, WAS, LPXN, CAPG, SH3BGR, NAPG |
| GO-BP | GO:0032060~bleb assembly | 3 | 0.006944 | 0.022761 | ROCK1, EMP2, MYLK |
| GO-BP | GO:0016477~cell migration | 10 | 0.023148 | 0.023212 | BTG1, SH3KBP1, FAT1, SNAI1, CDK1, SDC1, EMP2, TYK2, JAK2, JAK3 |
| GO-BP | GO:0033209~tumor necrosis factor-mediated signaling pathway | 8 | 0.018519 | 0.023676 | PYCARD, PSME1, JAK2, TNFRSF1B, PSMB8, PSMB10, TNFRSF21, PSMB9 |
| GO-BP | GO:0032956~regulation of actin cytoskeleton organization | 5 | 0.011574 | 0.025908 | ROCK1, ROCK2, ILK, ARHGAP18, SH3BGRL3 |
| GO-BP | GO:0031100~organ regeneration | 5 | 0.011574 | 0.025908 | CCNA2, CDK1, BAK1, MKI67, IGF2R |
| GO-BP | GO:0030855~epithelial cell differentiation | 6 | 0.013889 | 0.026338 | FZD1, GSTK1, TAGLN, CDK1, TPP1, CTSB |
| GO-BP | GO:0030336~negative regulation of cell migration | 7 | 0.016204 | 0.026799 | IFITM1, CLIC4, TPM1, PTPRJ, THY1, VCL, RHOB |
| GO-BP | GO:0097202~activation of cysteine-type endopeptidase activity | 3 | 0.006944 | 0.027383 | PYCARD, PERP, BAK1 |
| GO-BP | GO:0001886~endothelial cell morphogenesis | 3 | 0.006944 | 0.027383 | CLIC4, PECAM1, PLOD3 |
| GO-BP | GO:0050900~leukocyte migration | 8 | 0.018519 | 0.027768 | CD74, FCER1G, PIK3CA, ITGA4, ROCK1, PECAM1, ITGAX, C3AR1 |
| GO-BP | GO:0000226~microtubule cytoskeleton organization | 6 | 0.013889 | 0.027795 | SPECC1, SS18, DYNC1LI2, MAP2, CDK1, CAMSAP2 |
| GO-BP | GO:0038095~Fc-epsilon receptor signaling pathway | 10 | 0.023148 | 0.028152 | LAT2, NFKBIA, MAP3K1, FCER1G, PIK3CA, PSME1, PSMB8, PSMB10, PSMB9, SKP1 |
| GO-BP | GO:0090200~positive regulation of release of cytochrome c from mitochondria | 4 | 0.009259 | 0.028833 | PYCARD, PLAUR, PPIF, BAK1 |
| GO-BP | GO:0042752~regulation of circadian rhythm | 5 | 0.011574 | 0.029658 | TOP2A, PPP1CB, OPN3, ROCK2, PPP1CA |
| GO-BP | GO:0000281~mitotic cytokinesis | 4 | 0.009259 | 0.031602 | NUSAP1, CKAP2, KIF20A, CEP55 |
| GO-BP | GO:0050766~positive regulation of phagocytosis | 4 | 0.009259 | 0.031602 | PYCARD, C3, FCER1G, CYBA |
| GO-BP | GO:0007229~integrin-mediated signaling pathway | 7 | 0.016204 | 0.031945 | TYROBP, FCER1G, ITGA4, ITGAX, ILK, ITGA7, ITGAE |
| GO-BP | GO:1902287~semaphorin-plexin signaling pathway involved in axon guidance | 3 | 0.006944 | 0.032346 | PLXND1, SEMA3A, PLXNC1 |
| GO-BP | GO:0030334~regulation of cell migration | 6 | 0.013889 | 0.032472 | PLXND1, PECAM1, PLXNC1, THY1, VCL, RHOB |
| GO-BP | GO:0001934~positive regulation of protein phosphorylation | 8 | 0.018519 | 0.033527 | FZD1, C3, ROCK2, AKTIP, PLAUR, PRR5L, FAM20A, SENP2 |
| GO-BP | GO:0000209~protein polyubiquitination | 10 | 0.023148 | 0.033787 | RNF144B, DTX3L, HECTD2, PSME1, LNPEP, DTL, PSMB8, PSMB10, PSMB9, SKP1 |
| GO-BP | GO:0051437~positive regulation of ubiquitin-protein ligase activity involved in regulation of mitotic cell cycle transition | 6 | 0.013889 | 0.035846 | CDK1, PSME1, PSMB8, PSMB10, PSMB9, SKP1 |
| GO-BP | GO:0016236~macroautophagy | 6 | 0.013889 | 0.035846 | CLN3, PRKAB2, EI24, MFN1, TCIRG1, DYNLL2 |
| GO-BP | GO:0030036~actin cytoskeleton organization | 8 | 0.018519 | 0.037337 | SPECC1, SDCBP, ROCK1, RHOG, RAC2, TMSB10, PRKG1, PDLIM7 |
| GO-BP | GO:0051492~regulation of stress fiber assembly | 3 | 0.006944 | 0.037631 | PTGER4, ROCK1, ROCK2 |
| GO-BP | GO:0043518~negative regulation of DNA damage response, signal transduction by p53 class mediator | 3 | 0.006944 | 0.037631 | CD74, SNAI1, SENP2 |
| GO-BP | GO:0060334~regulation of interferon-gamma-mediated signaling pathway | 3 | 0.006944 | 0.037631 | IFNGR1, JAK2, PTPN2 |
| GO-BP | GO:0006888~ER to Golgi vesicle-mediated transport | 9 | 0.020833 | 0.039391 | BCAP31, DYNC1I2, DYNC1LI2, VAPB, ATL2, CTSZ, USO1, DYNLL2, CTSC |
| GO-BP | GO:0006954~inflammatory response | 16 | 0.037037 | 0.040643 | ECM1, PARP4, IGFBP4, NFAM1, CYBA, TNFRSF1B, PTGS1, C3, PYCARD, TLR1, SPP1, C3AR1, SDC1, CHI3L1, TNFRSF21, CCR2 |
| GO-BP | GO:0002223~stimulatory C-type lectin receptor signaling pathway | 7 | 0.016204 | 0.040804 | NFKBIA, FCER1G, PSME1, PSMB8, PSMB10, PSMB9, SKP1 |
| GO-BP | GO:0010875~positive regulation of cholesterol efflux | 3 | 0.006944 | 0.043219 | ABCA1, NFKBIA, PLTP |
| GO-BP | GO:0043101~purine-containing compound salvage | 3 | 0.006944 | 0.043219 | GMPR, AMPD3, ADA |
| GO-BP | GO:0001503~ossification | 6 | 0.013889 | 0.043222 | ECM1, IFITM1, TMEM119, SPP1, TAPT1, PDLIM7 |
| GO-BP | GO:0007018~microtubule-based movement | 6 | 0.013889 | 0.045198 | DYNC1I2, DYNC1LI2, KLC2, AP2S1, KIF1C, KIF20A |
| GO-BP | GO:1903012~positive regulation of bone development | 2 | 0.00463 | 0.047424 | TMEM119, TAPT1 |
| GO-BP | GO:0001798~positive regulation of type IIa hypersensitivity | 2 | 0.00463 | 0.047424 | C3, FCER1G |
| GO-BP | GO:0006955~immune response | 17 | 0.039352 | 0.047495 | PTGER4, IL32, CD74, LST1, WAS, SECTM1, SERPINB9, TNFRSF1B, HLA-F, C3, TLR1, ENPP1, IL7R, B2M, TNFRSF21, CTSC, CCR2 |
| GO-BP | GO:0070301~cellular response to hydrogen peroxide | 5 | 0.011574 | 0.047724 | TRPM2, CDK1, PPIF, AKR1B1, RHOB |
| GO-BP | GO:0097009~energy homeostasis | 3 | 0.006944 | 0.049093 | PIK3CA, PRCP, AMPD3 |
| GO-BP | GO:0006979~response to oxidative stress | 7 | 0.016204 | 0.049265 | TRPM2, BTG1, GPX1, MSRB3, PSIP1, ATOX1, PTGS1 |
| KEGG pathway | hsa04611:Platelet activation | 15 | 0.034722 | 1.65E-05 | PPP1R12A, FCER1G, ROCK1, ROCK2, ADCY3, GNAI1, MAPK13, PTGS1, PPP1CA, MYLK, PPP1CB, PLCB4, PIK3CA, ORAI1, PRKG1 |
| KEGG pathway | hsa05132:Salmonella infection | 12 | 0.027778 | 1.94E-05 | PYCARD, DYNC1I2, DYNC1LI2, KLC2, ROCK1, ROCK2, IFNGR1, ARPC1B, RHOG, WAS, RILP, MAPK13 |
| KEGG pathway | hsa04142:Lysosome | 13 | 0.030093 | 0.00015 | ATP6V0B, CTSZ, AP1B1, TCIRG1, IGF2R, PLA2G15, GALNS, CLN3, PPT1, TPP1, CTSD, CTSC, CTSB |
| KEGG pathway | hsa04810:Regulation of actin cytoskeleton | 17 | 0.039352 | 0.000285 | PPP1R12A, ROCK1, ITGA4, ROCK2, ARPC1B, WAS, ITGAE, GNG12, PPP1CA, MYLK, PPP1CB, SCIN, PIK3CA, ITGAX, RAC2, ITGA7, VCL |
| KEGG pathway | hsa04062:Chemokine signaling pathway | 15 | 0.034722 | 0.000771 | ROCK1, ROCK2, WAS, ADCY3, GNG12, GNAI1, CXCL16, NFKBIA, PLCB4, GNG5, PIK3CA, RAC2, JAK2, JAK3, CCR2 |
| KEGG pathway | hsa05152:Tuberculosis | 14 | 0.032407 | 0.001482 | CD74, ATP6V0B, FCER1G, IFNGR1, TCIRG1, LSP1, MAPK13, C3, TLR1, IRAK1, ITGAX, JAK2, CTSD, RAB5A |
| KEGG pathway | hsa04670:Leukocyte transendothelial migration | 11 | 0.025463 | 0.001495 | PIK3CA, ITGA4, ROCK1, ROCK2, PECAM1, RAC2, CYBA, THY1, GNAI1, VCL, MAPK13 |
| KEGG pathway | hsa04022:cGMP-PKG signaling pathway | 13 | 0.030093 | 0.00169 | PPP1R12A, ROCK1, ROCK2, ADCY3, GNAI1, PPP1CA, MYLK, PPP1CB, PLCB4, AGTR1, PPIF, SLC25A4, PRKG1 |
| KEGG pathway | hsa04270:Vascular smooth muscle contraction | 11 | 0.025463 | 0.001703 | PPP1CB, PPP1R12A, PLCB4, PRKCH, ROCK1, ROCK2, AGTR1, ADCY3, PRKG1, PPP1CA, MYLK |
| KEGG pathway | hsa05131:Shigellosis | 8 | 0.018519 | 0.00209 | NFKBIA, ROCK1, ROCK2, ARPC1B, RHOG, WAS, VCL, MAPK13 |
| KEGG pathway | hsa05140:Leishmaniasis | 8 | 0.018519 | 0.003788 | NFKBIA, C3, IRAK1, ITGA4, IFNGR1, CYBA, JAK2, MAPK13 |
| KEGG pathway | hsa04510:Focal adhesion | 14 | 0.032407 | 0.005561 | PPP1R12A, ROCK1, ITGA4, ROCK2, ILK, PARVB, PPP1CA, MYLK, PPP1CB, PIK3CA, SPP1, RAC2, ITGA7, VCL |
| KEGG pathway | hsa05142:Chagas disease (American trypanosomiasis) | 9 | 0.020833 | 0.009116 | NFKBIA, C3, C1QA, PLCB4, IRAK1, PIK3CA, IFNGR1, GNAI1, MAPK13 |
| KEGG pathway | hsa04924:Renin secretion | 7 | 0.016204 | 0.009199 | PTGER4, PLCB4, PDE1A, AGTR1, ORAI1, GNAI1, CTSB |
| KEGG pathway | hsa04921:Oxytocin signaling pathway | 11 | 0.025463 | 0.009996 | PPP1CB, TRPM2, PRKAB2, PPP1R12A, PLCB4, ROCK1, ROCK2, ADCY3, GNAI1, PPP1CA, MYLK |
| KEGG pathway | hsa05168:Herpes simplex infection | 12 | 0.027778 | 0.014652 | NFKBIA, PPP1CB, C3, IFNAR2, CD74, IFNGR1, CDK1, TYK2, JAK2, HLA-F, SKP1, PPP1CA |
| KEGG pathway | hsa04071:Sphingolipid signaling pathway | 9 | 0.020833 | 0.020317 | PLCB4, FCER1G, PIK3CA, ROCK1, ROCK2, RAC2, CTSD, GNAI1, MAPK13 |
| KEGG pathway | hsa04750:Inflammatory mediator regulation of TRP channels | 8 | 0.018519 | 0.020848 | PTGER4, PPP1CB, PLCB4, PRKCH, PIK3CA, ADCY3, MAPK13, PPP1CA |
| KEGG pathway | hsa05169:Epstein-Barr virus infection | 9 | 0.020833 | 0.022203 | NFKBIA, CCNA2, IRAK1, PIK3CA, NEDD4, TYK2, HLA-F, JAK3, MAPK13 |
| KEGG pathway | hsa04024:cAMP signaling pathway | 12 | 0.027778 | 0.024793 | NFKBIA, PPP1CB, GRIA2, PPP1R12A, PIK3CA, ROCK1, ROCK2, RAC2, ADCY3, ORAI1, GNAI1, PPP1CA |
| KEGG pathway | hsa04620:Toll-like receptor signaling pathway | 8 | 0.018519 | 0.030458 | NFKBIA, TLR1, IFNAR2, IRAK1, PIK3CA, SPP1, MAP3K8, MAPK13 |
| KEGG pathway | hsa04380:Osteoclast differentiation | 9 | 0.020833 | 0.032234 | NFKBIA, IFNAR2, SPI1, TYROBP, PIK3CA, IFNGR1, CYBA, TYK2, MAPK13 |
| KEGG pathway | hsa04962:Vasopressin-regulated water reabsorption | 5 | 0.011574 | 0.035168 | DYNC1I2, DYNC1LI2, ADCY3, RAB5A, DYNLL2 |
| KEGG pathway | hsa05145:Toxoplasmosis | 8 | 0.018519 | 0.036229 | NFKBIA, IRAK1, IFNGR1, PPIF, TYK2, JAK2, GNAI1, MAPK13 |
| KEGG pathway | hsa04261:Adrenergic signaling in cardiomyocytes | 9 | 0.020833 | 0.041914 | PPP1CB, PLCB4, TPM2, TPM1, AGTR1, ADCY3, GNAI1, MAPK13, PPP1CA |
