# Supplementary material for: Analysis of genes and underlying mechanisms involved in foam cells formation and atherosclerosis development
Source: PeerJ. 2020 Nov 17;8:e10336. doi: 10.7717/peerj.10336 (PMC7678445; doi:10.7717/peerj.10336)
Supplement: Table S2 [file peerj-08-10336-s002.doc]

Table S2 GO-BP and KEGG pathway analysis of M-FC-related genes

| Category | Term | Count | PValue | Genes |
| --- | --- | --- | --- | --- |
| GO-BP | GO:0010887~negative regulation of cholesterol storage | 4 | 1.56E-06 | PPARG, ABCA1, ABCG1, NR1H3 |
| GO-BP | GO:0010745~negative regulation of macrophage derived foam cell differentiation | 4 | 2.19E-05 | PPARG, ABCA1, ABCG1, NR1H3 |
| GO-BP | GO:0007165~signal transduction | 16 | 1.17E-04 | TNFRSF21, RYK, PPARG, NLRP3, OCRL, CD74, PLAUR, ARHGAP4, TNFSF10, TNFRSF11A, EVI2A, CD33, INPP5D, CDC42EP3, ARHGAP9, CSF1R |
| GO-BP | GO:0033993~response to lipid | 3 | 5.13E-04 | CD36, PPARG, ABCG1 |
| GO-BP | GO:0016064~immunoglobulin mediated immune response | 3 | 1.00E-03 | INPP5D, CD74, TLR8 |
| GO-BP | GO:0010875~positive regulation of cholesterol efflux | 3 | 0.00164 | ABCA1, ABCG1, NR1H3 |
| GO-BP | GO:0006955~immune response | 8 | 0.002325 | TNFRSF21, TNFSF10, LST1, CD36, CTSC, HLA-DPA1, FTH1, CD74 |
| GO-BP | GO:0055098~response to low-density lipoprotein particle | 2 | 0.012986 | PPARG, ABCA1 |
| GO-BP | GO:0071222~cellular response to lipopolysaccharide | 4 | 0.013119 | CD36, ABCA1, NLRP3, NR1H3 |
| GO-BP | GO:0060336~negative regulation of interferon-gamma-mediated signaling pathway | 2 | 0.021551 | PPARG, NR1H3 |
| GO-BP | GO:0002830~positive regulation of type 2 immune response | 2 | 0.025806 | NLRP3, CD74 |
| GO-BP | GO:0032367~intracellular cholesterol transport | 2 | 0.025806 | ABCA1, ABCG1 |
| GO-BP | GO:0050852~T cell receptor signaling pathway | 4 | 0.026648 | TNFRSF21, HLA-DPA1, INPP5D, PAK1 |
| GO-BP | GO:0002250~adaptive immune response | 4 | 0.026648 | TNFRSF21, LAT2, TNFRSF11A, CLEC4A |
| GO-BP | GO:0060907~positive regulation of macrophage cytokine production | 2 | 0.030042 | CD36, CD74 |
| GO-BP | GO:0042632~cholesterol homeostasis | 3 | 0.0316 | ABCA1, ABCG1, NR1H3 |
| GO-BP | GO:0050702~interleukin-1 beta secretion | 2 | 0.034261 | CD36, ABCA1 |
| GO-BP | GO:0002674~negative regulation of acute inflammatory response | 2 | 0.034261 | PPARG, NLRP3 |
| GO-BP | GO:0060100~positive regulation of phagocytosis, engulfment | 2 | 0.034261 | CD36, PPARG |
| GO-BP | GO:0006952~defense response | 3 | 0.03437 | CLEC1A, NLRP3, CD74 |
| GO-BP | GO:0071300~cellular response to retinoic acid | 3 | 0.037231 | SERPINF1, PPARG, ABCA1 |
| GO-BP | GO:0055091~phospholipid homeostasis | 2 | 0.038461 | ABCA1, ABCG1 |
| GO-BP | GO:0045087~innate immune response | 6 | 0.039115 | PPARG, CLEC4A, APOBEC3G, NLRP3, TLR8, CSF1R |
| GO-BP | GO:0070374~positive regulation of ERK1 and ERK2 cascade | 4 | 0.040707 | CD36, PDGFC, CD74, CSF1R |
| GO-BP | GO:0007596~blood coagulation | 4 | 0.046081 | CD36, F13A1, PDGFC, PLAUR |
| GO-BP | GO:0050728~negative regulation of inflammatory response | 3 | 0.046333 | SERPINF1, NLRP3, NR1H3 |
| GO-BP | GO:0042127~regulation of cell proliferation | 4 | 0.046699 | TNFRSF21, HHEX, DHCR7, PKD2 |
| GO-BP | GO:0034374~low-density lipoprotein particle remodeling | 2 | 0.046808 | PLA2G7, ABCG1 |
| GO-BP | GO:0007155~cell adhesion | 6 | 0.04936 | CD36, PCDHB6, TLN2, CD33, SIGLEC7, CLEC4A |
| GO-BP | GO:0050731~positive regulation of peptidyl-tyrosine phosphorylation | 3 | 0.049532 | CD36, HCLS1, CD74 |
| KEGG pathway | hsa03320:PPAR signaling pathway | 4 | 0.005012 | CD36, CYP27A1, PPARG, NR1H3 |
